# A Portable System to Monitor Saliva Conductivity for Dehydration Diagnosis and Kidney Healthcare

**DOI:** 10.1038/s41598-019-51463-8

**Published:** 2019-10-14

**Authors:** Yen-Pei Lu, Jo-Wen Huang, I-Neng Lee, Rui-Cian Weng, Ming-Yu Lin, Jen-Tsung Yang, Chih-Ting Lin

**Affiliations:** 1 Taiwan Instrument Research Institute, National Applied Research Laboratories, 30261 Hsinchu, Taiwan; 2 Department of Neurosurgery, Chang-Gung Memorial Hospital, 61363 Chia-Yi, Taiwan; 3 Department of Medical Research, Chang Gung Memorial Hospital, Chiayi, 61363 Taiwan; 4 College of Medicine, Chang Gung University, Tao-Yuan, 33302 Taiwan; 5 Graduate Institute of Electrical Engineering, National Taiwan University, 10617 Taipei, Taiwan

**Keywords:** Diagnostic markers, Laboratory techniques and procedures, Biomedical engineering

## Abstract

Chronic kidney disease (CKD) has become a major issue in long-term healthcare. It is caused by recurrent kidney injury, which is possible induced by dehydration and heat stress. Therefore, it is important to access the dehydration diagnosis on fields. Conventional instruments for assessing dehydration from blood and urine samples are expensive and time-consuming. These disadvantages limit their applications in high-risk groups susceptible to kidney disease. To address this unmet need, this study presents a portable miniaturized device for dehydration diagnosis with clinical saliva samples. With co-plane coating-free gold electrodes, the dehydration diagnosis was achieved with a saliva specimen at low volumes (50–500 μL). To examine the characteristics, the developed device was assessed by using standard conductivity solutions and the examined variation was <5%. To validate the use for field applications, saliva samples were measured by the developed device and the measured results were compared with standard markers of serum osmolality (N = 30). These data indicate that the measured saliva conductivity is consistent with serum osmolality. And it shows significant difference between healthy adults and healthy farmers (*p* < 0.05), who typically suffer high risks of CKD. Based on this work, the proposed device and measurement offer a useful method to diagnosis dehydrations and indicate possible potential for CKD.

## Introduction

Chronic kidney disease (CKD) is caused by persistent damage to kidneys; it can worsen over time and lead to the progressive kidney function loss. The kidneys may stop working in the late stages of CKD, eventually progressing to end-stage kidney failure requiring hemodialysis or kidney transplantation. Globally, 10%–15% of the adult population are predicted to suffer CKD and annual costs for an end-stage patient range between US$35,000 and US$100,000^1^. In the past decade, clinicians in Sri Lanka, Central America, and India have discovered that in their rural agricultural communities, a high proportion of patients were suffering from CKD. They were predominantly paddy farmers and sugarcane workers exposed to long-term heat stress and chronic dehydration, which contribute to heat-stress nephropathy and CKD. Evidence indicates that chronic dehydration is one of the most likely causes of CKD of unknown aetiology in farmers. Different methods were proposed to evaluate the association between CKD and farmers^2–5^. These previous results have discovered related variations in the hydration and kidney function biomarkers among sugarcane workers or paddy farmers.

Dehydration, a consequence of excess depletion of body water, is frequently associated with abnormalities in electrolyte balance; therefore, osmolality can enable the detection of dehydration through the influence of its volume-mediated aspects. Blood plasma, serum, and urine osmolality have been proven for evaluating dehydration, e.g. hematological osmolality is the index of hydration status. Human body is in euhydrated condition as plasma osmolality ≤290 mOsm/Kg^6^, serum osmolality ≤295 mOsm/Kg^7^, or urine osmolality ≤700 mOsm/Kg^6^. In addition, the correlation between saliva osmolality and body mass change have been also investigated. Therefore, saliva osmolality is an effectively diagnosed biomarker during active dehydration (sensitivity = 86%; specificity = 91%) compared with serum osmolality, urine osmolality, urine volume, and urine-specific gravity^8^. However, the equipment required for osmolality measurements is expensive, time-consuming, and require large sample sizes. Therefore, developing a simple and portable device for detecting dehydration is crucial.

In the past decade, saliva has been investigated as a valuable fluid for detecting potential biomarkers in various clinical diagnoses^9–12^. Several characteristics including salivary flow rate, salivary osmolality, and concentration of salivary electrolytes have been reported to be related to dehydration or CKD^13,14^. The salivary flow rate can be used to estimate the amount of saliva secreted from the salivary glands, which cease secretion to conserve water during dehydration. In this estimation, only the volume of saliva and collection period are required. In a study, the mean (±standard deviation) salivary flow rate between smokers and nonsmokers was 0.20 (±0.05) and 6.30 (±0.36) mL/min, respectively, which was a statistically significant difference^15^. However, changes in salivary flow are unreliable because they are affected by various factors, including body posture, smoking, and lighting conditions. Therefore, this method varies significantly between individuals^16,17^. On the other hand, the measurement of electrolytes using ion-selective electrodes has been widely investigated in clinical analyses^18,19^, including that of saliva content^20^. Although the required equipment is relatively inexpensive and a straightforward technique, the composition of electrolytes varies with individuals, such as circadian rhythm. This is also a key disadvantage explaining the reason that counting a single ion alone or minority ions is not an ideal marker for assessing dehydration. For instance, chloride and sodium are the major ions secreted by saliva gland acinar cells. The concentration of salivary sodium reaches its maximal level early in the morning, and both sodium and chloride ions have a minimum concentration in the afternoon^16,21^. Since CKD has a high mortality rate, restricting patients’ participation in the workforce and affecting their quality of life, it is of importance to develop an effective and robust method to monitor dehydration for the high-risk populations before they develop CKD.

In this paper, we propose a robust device to quantitively detect hydration status by analyzing saliva conductivity (an osmolality equivalent) with clinical samples. Compared with haematological and urinary markers in the clinical detection of dehydration, in real applications, saliva may be more appropriate for farmers who frequently experience dehydration or heat stress^22,23^. To examine the developed device’s performance, sodium chloride (NaCl) solution was used as the standard samples. To validate the potential of the developed device for use in clinical settings, furthermore, several groups of human saliva specimens including healthy adults, healthy farmers, and patients with CKD were collected and evaluated. This clinical validation demonstrates potentials of the developed device in field applications.

## Results

**Figure 1:**
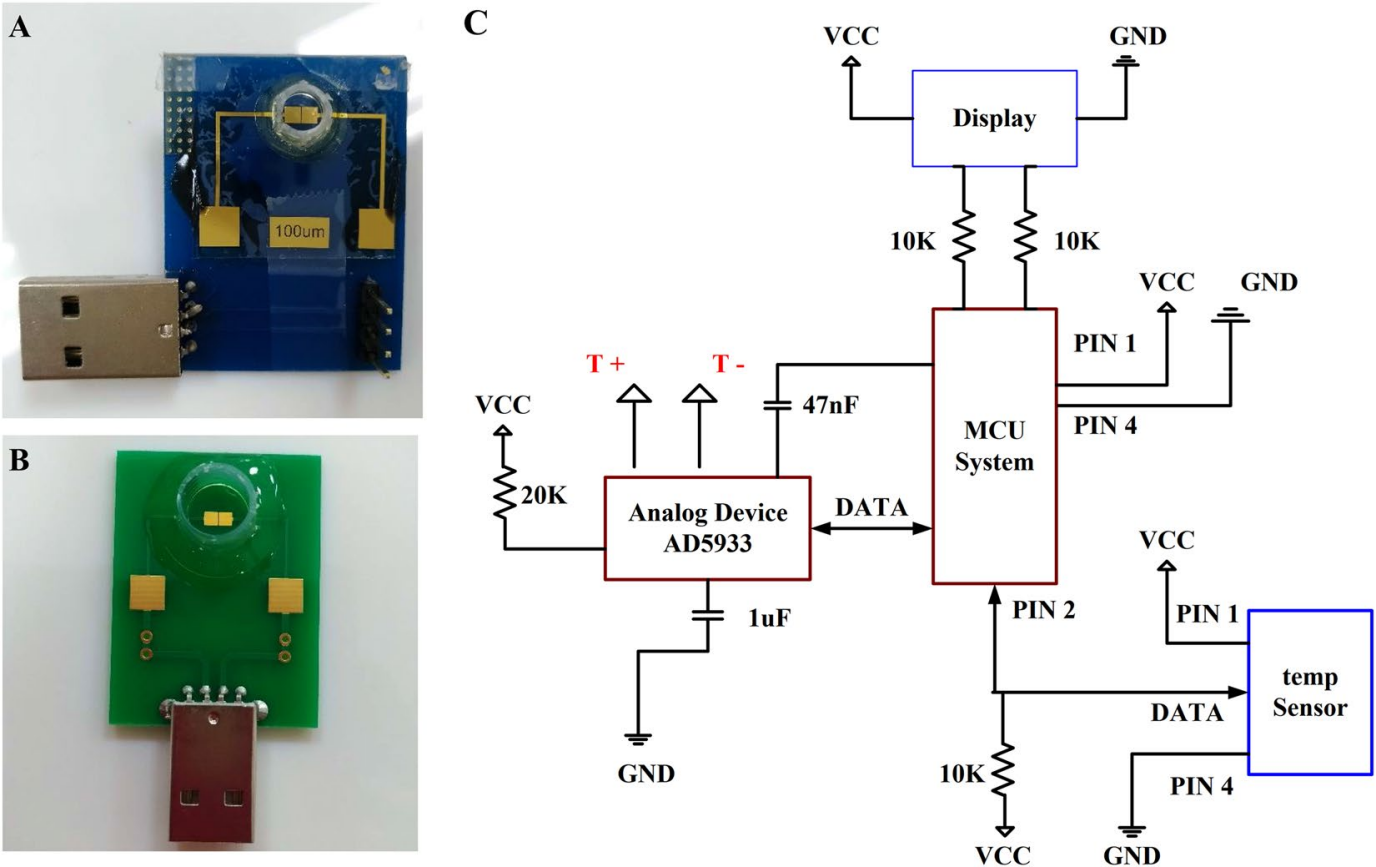
(**A**) Laboratory-made miniaturized Au electrode on glass substrate. (**B**) PCB-layout Au-electrode device. (**C**) Basic concept of the portable system design.

Electrode and portable system characterization. This study develops a home-use sensing device capable of measuring saliva conductivity through the assessment of bioelectrical signals. Current commercial technologies measure bioanalytes in rather large sample volumes, e.g. 3–10 mL of biological fluid. This required volume is inconvenient for clinical sampling and reduces patients’ willingness to comply. To address this problem, the developed device has a miniaturized (2 × 2 mm^2^) Au electrode (Fig. 1A,B). The miniaturization of electrodes reduces its sample-contact area and the amount of saliva required for a test. Therefore, this electrode design minimizes the required sample size to 50–500 μL. The developed device measures the electrical admittance between the micro-electrodes. The admittance variation is directly correlated to the ionic concentration of the electrolyte. Utilizing the built-in front-end amplifier of AD5933 (Analog Devices), as illustrated in Fig. 1C, the input signal can be received by its 12-bit analog-to-digital converter (ADC) through an inverting buffer amplifier. Based on the function designed in AD5933, the real-part and imaginary-part of the measured impedance can be obtained through I2C offered within AD5933. In the datasheet of AD5933, it specifies that it has a good signal-to-noise ratio of 60 dB. With preliminary examinations, therefore, a pre-amp is not necessary to be employed in the developed portable system. And the measurement system can be implemented with 1-Vpp and 1-kHz sine waves via AD5933, which has 1 MHz sampling rate and 12-bit analog-to-digital converter (ADC). The detecting electrode was externally connected to the portable sensing system through USB port. Since the sample volume is small, e.g. 50 μL, it was assumed the sample temperature achieves equilibrium with its ambient temperature within a short time period. An ambient temperature sensor was installed on the reader to compensate the temperature effect. Then the obtained signals are processed within a micro control unit (MCU) through a temperature-feedback block (Temp Sensor). This can improve the accuracy of admittance calculation. In the clinical study, this miniaturized system with printed-circuit board (PCB) devices was employed to measure the clinical saliva samples (IRB No. 201801708B0).

### Examination of designed electrodes’ sensitivity performance

**Figure 2:**
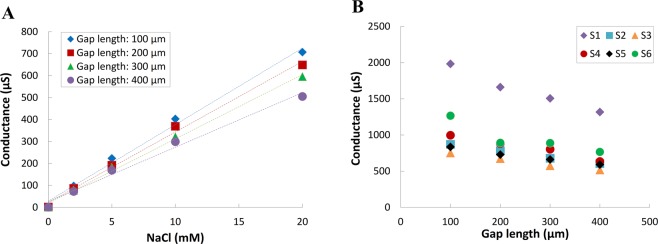
Sensitivity comparison of the four gap lengths between electrodes in (**A**) NaCl solution (0–20 mM) and (**B**) six saliva specimens from healthy adults.

To optimize the sensitivity of the developed micro-electrode, different gap length, i.e. 100, 200, 300, and 400 μm, between the Au microelectrodes on glass substrates were implemented. The performance of these different-gap microelectrodes was evaluated using sodium chloride (NaCl) as an analytic electrolyte at concentrations of 0, 2, 5, 10, and 20 mM. As shown by the results in Fig. 2A, for the 100, 200, 300, and 400 μm gap lengths, the ionic sensitivity slopes are 0.035, 0.032, 0.029, and 0.025, respectively; and the R2 are 0.993, 0.995, 0.997, and 0.988, respectively. According to the linearized sensitivity for impedance measurement, furthermore, impedance can be represented by Z = R + *j*X in a rectangular coordinate. The resistance R is the real part of the impedance, the reactance X is the imaginary part of the impedance, Z is the impedance, *j* is the imaginary unit, and θ is the phase of the impedance^24^. Based on the vector form of Coulomb’s law, the smaller gap of electrodes will have a greater slope of theoretically verified impedance, therefore, will have the better-received signal strength and sensitivity^25^. Based on these results, the 100-μm gap electrode has the highest sensitivity. Furthermore, the feasibility of the fabricated device in saliva measurement was also examined as shown in Fig. 2B. In this examination, six saliva samples from healthy adults were employed. Figure 2B also shows the highest sensor response can be achieved by the 100-μm gap electrode. In this examination, the saliva conductance of well-hydrated people was examined in the range from approximately 752.5 μS to 1983.1 μS. Therefore, the 100-μm gap length device is used for following clinical examinations.

### Evaluation of particle interferences

**Figure 3:**
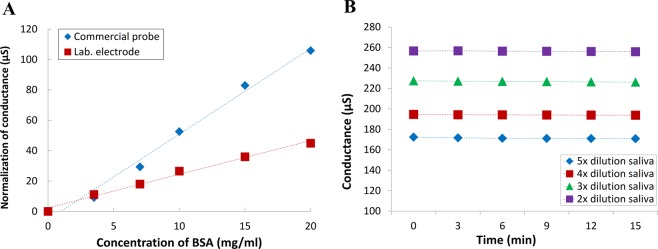
Stable performance evaluation of the electrodes: (**A**) Two types of electrode were used for detection in 5 mM NaCl solution spiked with BSA (0–20 mg/mL); (**B**) a 15-minute measurement of a human saliva sample with different dilution factor by using microfabricated electrodes.

Oral fluid is complex. It contains proteins secreted by salivary gland cells to create viscoelasticity. In addition, exogenous sources such as food debris and microbiota are suspending in the solution or adhere to the surface of electrodes. These materials cannot be neglected since its charged surface may interfere the analysis of electrical signals^26^. In this study, we proposed a method to conquer this problem by non-coating co-planar electrode design. Since signal follows the minimum impedance path, the major signal component passes through the path at the gap-edge of the electrode^27^. Therefore, the area near the gap between two electrodes has a relatively large ratio of electric field variables^28^. At the same time, most of particle interferences falls at the top of the electrode. As a consequence, the proposed co-planar electrode design can reduce the interference induced by food debris and microbiota. At the same time, most of particle interferences falls at the top of the electrode. As a consequence, the proposed co-planar electrode design can reduce the interference induced by food debris and microbiota. To examine this proposed design, an LCR meter was used to facilitate comparisons between the proposed electrodes, e.g. micro-fabricated electrode on a glass substrate (laboratory-made electrode) and a commercial conductivity-measurement probe. Figure 3A illustrates the measured conductance from different electrodes with the test samples. These measurements performed in a 5 mM NaCl electrolyte solution with different concentration of bovine serum albumin (BSA) interference molecules. Figure 3A shows that a clear correlation to BSA concentration can be observed in the measurement of the commercial probe. Compared with the commercial probe, the designed co-planar microelectrode has less interference from BSA. In quantitative evaluations, the co-planar microelectrode has a slope of 2.245 μS/(mg/mL). By contrast, the commercial probe has a slope of 5.295 μS/(mg/mL). This clearly shows that the proposed co-planar electrode design has less vulnerability to interference proteins than the commercial electrode does. To further examine the resistance of particle interference of the proposed micro-electrode design in clinical saliva specimens, different dilution ratio of a saliva sample to DI water were employed for 15-minute measurements. With different dilution ratio, the interference-particle concentrations are also different. Since debris accumulated on the surface with time, this measurement can be used to evaluate the interference-particle effect of the developed co-planar microfabricated electrode. The experimental results can be shown in Fig. 3B. It plots the variations for the human saliva samples with different viscosities over the course of 15 min. Four dilution ratio of saliva were measured, namely 2×, 3×, 4×, and 5× v/v diluted saliva. The measured variations are 0.9%, 0.4%, 0.5%, and 0.3% respectively. A constant signal can be observed during the 15-minute measurement. This indicates that the developed device has a good resistance to interference-particle accumulations. The steady conductance measurement demonstrates that the electrode could be applied in saliva samples and maintain a stable signal.

### Comparison of performance of different fabricated electrodes and measurement system

**Figure 4:**
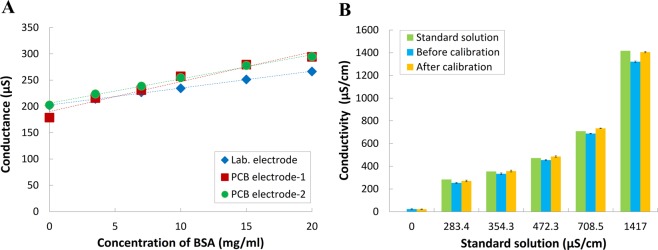
Performance in an interference test with protein factor. (**A**) The microfabricated electrodes were compared with two PCB electrodes using 5 mM NaCl solution spiked with BSA interference solution (0–20 mg/mL). (**B**) Six concentrations of standard NaCl conductivity solutions measured using the portable sensing system and PCB electrode.

Microfabricated electrodes have good performances in interference measurements. However, it is not the most economic fabrication process for the application of on-field saliva tests. By contrast, PCB techniques have been developed for mass-production electrical systems with resolution of 100 μm. As a consequence, it is intriguing to employ the PCB manufacturing technologies to fabricate the co-planar electrodes with 100 μm gap. To assess the performance of PCB electrodes by an LCR meter, Fig. 4A demonstrates the measurement of a 5 mM NaCl solution with the presence of different concentration of BSA. The conductance measurement results of the PCB and laboratory-made electrodes has *p* values of 0.62 and 0.36, respectively. Therefore, the difference between these two fabricated devices is nonsignificant. Moreover, the correlation coefficient is 0.99, which shows high consistency. These results indicate that commercially manufactured PCB electrodes would be as effective as a laboratory-made electrode in measuring high-protein samples. Therefore, our electrodes can be manufactured using standard commercial PCB production techniques, which offer the advantages of a low-volume requirement, disposability, and reliable sensing. In addition, the temperature effect of saliva samples was also examined as 1.9%/°C. Since the required saliva sample is small in volume in the developed device, the temperature effect should be further mitigated. To address this concern, an additional temperature sensor is implemented and enables direct monitoring of temperature in our portable system. This can mitigate the concerns of samples being affected by temperature and minimize reading errors resulting from measuring samples under different environmental temperatures. This is a feature that sets our portable system apart from other environmental monitoring methods, as our design exercises temperature control to obtain optimal results. To assess whether our self-developed portable sensing system could be as effective in measurements as large laboratory-based equipment, we tested the conductivity of a series of standard NaCl conductivity solutions as shown in Fig. 4B. The standard solutions’ 25 °C electrical conductivity given by manufacturer were 283.4, 354.3, 472.3, 708.5 and 1417 μS/cm, respectively. Before temperature calibration process, the measurements obtained from the developed portable system has difference of 10.46, 5.84, 3.81, 2.86, and 6.78, respectively. After temperature calibration, the difference can be reduced to 4.43, 0.97, 2.87, 3.59, and 0.84, respectively. Because the percent deviations after temperature calibration were all <5%, the portable sensing system was deemed sufficiently accurate.

### Implementation of saliva sensor in clinical evaluation of hydration status

**Figure 5:**
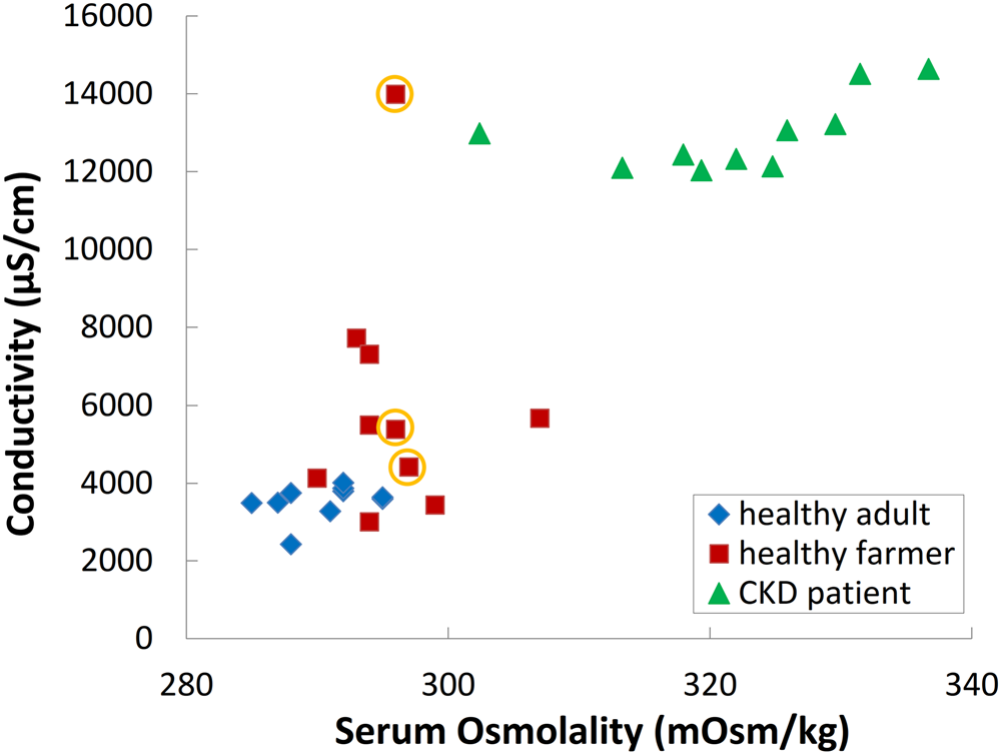
Relationship between saliva conductivity and serum osmolality; distribution of the three populations (healthy adults [N = 10], healthy farmers [N = 10], and patients with CKD [N = 10]).

Relevant studies have reported saliva parameters including salivary flow rate, osmolality, and total protein concentration to be markers of hydration status^29^. The developed device and portable system must be validated to correlate strongly with blood osmolality or urine gravity, which are commonly known as the hydration index, to quantify changes in a clinical setting^6^. Researches on assessing hydration levels using saliva conductivity has been established at the proof-of-concept level with a functioning sensor and compared with serum osmolality. Clinical trials (IRB 201801708B0) were performed on health examination groups (healthy adults and healthy farmers) as well as dialysis patients with CKD through monitoring their serum osmolality and saliva conductivity. Figure 5 shows that the mean (±standard deviation) saliva conductivity readings of the healthy adults, healthy farmers, and patients with CKD are 3531.5 (±441.7), 6050.7 (±3174.8), and 12,945.0 (±958.9) μS/cm, respectively. At the same time, the mean serum osmolality readings of the healthy adults, healthy farmers, and patients with CKD are 290.5 (±3.4), 296.0 (±4.6), and 322.4 (±9.8) mOsm/kg, respectively. The distributions of three groups are substantially skewed and the correlations are calculated between saliva conductivity and serum osmolality (*r* = 0.85). Compared with the healthy adults, saliva conductivity levels increase significantly in the health farmers (*p* = 0.003) and patients with CKD (*p* < 0.001). Among them, the saliva conductivity readings of the patients with CKD exhibits significant differences compared with those of the farmers (*p* < 0.001). All serum osmolality readings in the patients with CKD are above the threshold of 295 mOsm/Kg, which is considered the upper limit of dehydration. On the other hand, all osmolality readings of the healthy adults are ≤ 295 mOsm/Kg, and 50% of those in the farmers were >295 mOsm/Kg. These results indicated that hydration status for healthy adults, healthy farmers, and patients with CKD can be diagnosed by the developed saliva examination method.

## Discussion

Although many standard methods, such as osmolality, urine gravity, BUN, and creatinine ratio are useful for assessing dehydration and kidney function, collecting clinical blood or urine samples is difficult and acquiring testing data immediately is not possible. By contrast, saliva specimens can be collected noninvasively without specific equipment or time and environmental restrictions. However, there is no effective point-of-care instrument for the quick and convenient detection of dehydration and latent kidney diseases using saliva samples. Therefore, in this study, we developed a portable sensing system featuring a disposable PCB electrode that can detect hydration status in saliva specimens. The electrodes use gold as the conductive material because of its high biocompatibility, excellent stability, and reusability. Compared with the commercial probe, the developed miniaturized co-planar gold electrodes could function with a small sample volume of 50 μL instead of 3 mL. In a clinical setting, this is expected to considerably reduce the difficulty in sample acquisition and enhance patients’ willingness to comply. Because a slight change in a biomarker of a sample can drastically change its implication, testing sensitivity is crucial. For this purpose, four gap lengths (100, 200, 300, and 400 μm) co-planar electrodes were designed and examined their sensing sensitivity by using NaCl solution and saliva specimens. As shown in Fig. 2A, all four gap lengths had high correlation coefficients between the signal conductance and NaCl concentration (correlation coefficient = 0.99). The maximum sensitivity can be obtained by the electrode with gap length of 100 μm (0.035). It should be noted that the sensitivity could be enhanced by further reducing the gap length. However, the scale of hundreds μm has major advantages in manufacturing. Furthermore, saliva samples of six healthy adults were collected for conductivity analysis as shown in Fig. 2B. This experimental result reveals that 100-μm device obtained a maximum difference at 123.1 μS. By contrast, 400-μm device got a maximum difference at 80.1 μS. Therefore, the gap length of 100 μm was deemed to provide the gold electrodes with the good sensitivity to conductive substances of saliva, and thus it was adopted for the gold electrodes in the portable device and associated system design.

A relevant study demonstrated a conductivity detection of urine (an osmolality equivalent) with commercial instruments and its feasibility in evaluating the hydration status of athletes^30^. The mean urinary protein concentration is approximately 0.1–0.2 mg/mL^31^, whereas the mean concentration of human salivary proteins is approximately 0.72–3.72 mg/mL^32,33^. For instance, saliva comprises 99% water and 1% inorganic substances, such as sodium, potassium, chlorides, and bicarbonates, as well as organic substances, such as amylases, lysozymes, immunoglobulins, peroxidases, and agglutinins. The higher concentration of organic compounds in saliva leads to a distinct problem when measuring salivary conductance parameters because proteins adhere to the electrodes. This adhesion decreases electrode sensitivity for detecting electrolytes. To conquer this problem, surface modifications, such as zwitterionic molecules, PEG polymers, and antibiofouling polymers, have been studied to create an antifouling coating^7^. However, these approaches have limitations, e.g. production difficulty, multi-step processes, and poor mechanical properties. To measure the conductivity of high-protein samples, instead of complicated modifications, a non-coating co-planar electrode design is proposed and microfabricated in this work. Utilizing a standard NaCl solution with 5 mM concentration, both microfabricated electrodes on glass substrates and commercial electrodes were used to measure the conductance with different BSA concentration. Figure 3A shows a less BSA interference in the microfabricated electrodes than that in the commercial electrodes. This is attributed to the fact that electrode surface is more vulnerable to protein adhesions than electrode edge is. Since electrical signal path mainly at electrode edges in the design of co-planar microelectrode, therefore, the developed design provides a better sensing performance than the commercial electrode does in high-protein samples. On the other hand, the particle accumulation effect is also examined by employing different dilute ratio, i.e. 2x, 3x, 4x, and 5x diluted with DI water, of a saliva sample as shown in Fig. 3B. This shows the variations are <1% in all concentration and it suggests that the proposed electrode design should be useful for conductivity measurement with minimizing interference from debris and nasal secretions.

After examinations of the effectiveness of designed electrode, the ease and cost of manufacturing methods are considered to promote on-field applications. Compared with microfabrications, the PCB technology has advantages of cost and manufacturing. To validate the use of PCB electrodes, the conductance measurement of different concentration of BSA in a standard 5 mM NaCl solution was employed in both microfabricated electrode and PCB electrode as shown in Fig. 3A. The result reveals that the performance of the mass-produced PCB electrode was consistent with that of the microfabricated electrode, attaining a correlation of 0.99. As a consequence, the proposed saliva conductivity measurement can be implemented by standard PCB production techniques, which offer advantages of low cost and reliable sensing. Furthermore, a portable sensing system with a built-in temperature correction is developed based on the architecture shown in Fig. 1C. Based on the developed co-planar electrode and the portable sensing system, Fig. 4B demonstrates comparisons between standard solution conductivity (0–1417 μS/cm) and the measured value obtained from the portable system. It shows the developed portable sensing system is comparable with the given standard-solution conductivity and all the readings differences are less than 5%. Therefore, the developed system can be adopted and subjected to following clinical tests along with proposed electrode design.

Clinically, dehydration assessment is most commonly performed with blood or urine samples, with biomarkers such as serum osmolality, BUN/creatinine ratio, and urine gravity. These are widely recognized as the ‘gold standard’^2,6^. This study employed the proposed portable system and electrode for clinical tests, investigating whether saliva conductivity was positively correlated with these golden standards as shown in Fig. 5. A total of 30 participants were recruited and divided into three groups, namely healthy adults (N = 10), healthy farmers (N = 10), patients with CKD (N = 10). The saliva conductivity readings of the participants were compared with their serum osmolality and BUN/creatinine ratio to determine whether saliva conductivity could serve as an indicator for kidney diseases. This clinical test demonstrates that serum osmolality exhibited a consistency with that of saliva conductivity (*r* = 0.85). Compared with the healthy adults, furthermore, the healthy farmers had significantly higher mean saliva conductivity and serum osmolality when they finished work (*p* = 0.003). At the same time, participants of the CKD group exhibited significantly higher saliva conductivity and serum osmolality mean values compared with the healthy adults group (*p* < 0.001). The saliva conductivity readings of participants in the healthy adults group were all lower than the predetermined cutoff (4267 μS/cm) and their serum osmolality readings were also ≤295 mOsm/kg. Regarding patients with CKD, their saliva conductivity readings were higher than the cutoff and their serum osmolality readings were >295 mOsm/kg. According to the result of ROC curve, setting an osmolality of 295 mOsm/kg and saliva conductivity of 4267 μS/cm as the dehydration cutoff values. The diagnosis sensitivity was 93.3%, specificity was 80%, accuracy was 86.7%, false positive rate was 6.7%, and false negative rate was 20%. This proposes a 91.1% chance that saliva conductivity will correctly distinguish a dehydration status with an outstanding discriminating ability (*p* < 0.001). Furthermore, three participants (marked in yellow circles in Fig. 5) in the healthy farmers group had a BUN/creatinine ratio larger than 20. This suggests that they might have developed kidney diseases (their saliva conductivity and serum osmolality were higher than the cutoff). These results indicated that our method has the potential to detect CKD. Moreover, the testing results verify that a higher percentage of farmers are in a dehydrated state, which may increase their risk of kidney diseases. This finding is in agreement with the literature^2,3^.

In conclusion, our research identified a reliable and highly convenient diagnostic technique for detecting dehydration at home or on the field. The required low-volume sample of 50 μL results in ease of sample collection without a professional technician or specific environment. Based on the proposal electrode design and portable sensing system, the developed technique can be used to diagnose dehydrations and risk of CKD. The developed portable system has the potential to provide farmers with dependable information concerning their hydration condition via a small volume of saliva. It greatly increases the willingness of potential patients to comply, and thus also to monitor their daily hydration status. Therefore, it provides a real-time and convenient method for achieving CKD prevention. Because this is a pilot study to confirm the feasibility of dehydration testing, the hypothesis requires further confirmations in a larger-scale patient groups and the dehydration cutoff of saliva specimens must be clarified.

## Methods

### Electrode fabrication and experimental setup

Figure 1 presents the fabricated electrode which adopts co-planar configuration. The size of the fabricated electrode is 2 × 2-mm^2^ size. The microfabricated electrode was fabricated with evaporated Cr/Au (10 nm/100 nm) on a glass substrate. And the microfabricated electrodes were wired bond to a PCB for signal measurements. On the other hand, the PCB electrode was made on the FR4 board material. The electrode was made by applying electroless nickel immersion gold. The thickness of the gold is 75 nm and the gap length is 100 μm. Based on the implemented electrode, the conductivity can be measured. The following formula was applied for the conversion between conductance (S) and conductivity (μs/cm):

1 $${\rm{Conductivity}}\,({\rm{\mu }}s/{\rm{cm}})={\rm{conductance}}\,({\rm{S}})/{\rm{gap}}\,{\rm{length}}\,({\rm{cm}}).$$

To have the designed measurements, the sample fluidic well was cut from 5 mL syringes and fixed to the glass or PCB surface on the area of electrodes. For the standard NaCl solution, NaCl (Sigma, USA) was diluted in DI water and prepared at concentrations of 0, 2, 5, 10, and 20 mM. And solution conductance was measured using an LCR meter (HIOKI IM3533, Japan) and the sample volume was 50 μL of NaCl solution. All test tubes were prepared in triplicate. For the data analysis, the *p* value and correlation coefficient in this study were determined using the statistical Students’s t tests and a *p* value < 0.05 represented statistically significant results.

### Portable sensing system

The portable sensing system consists of an integrated circuit (AD5933), a micro control unit (MCU), an analogue-to-digital converter (ADC), and a liquid crystal display (LCD) to show electric signals of saliva samples. The circuit design follows a systematic architecture as presented in Fig. 1C. The system is designed for miniaturized electronic devices and used to conduct tests within an operating frequency range of kHz. To accomplish the admittance measurement, AD5933 (Analog Devices, USA) is employed. The measurement requires the gain factor (GF), and the equation for GF can be applied to calibrate electrical conductivity. The equation can be presented in Eq. (2). For example, if a 2 K precision resistor is used as the target for a test, the magnitude can be obtained using a direct digital synthesizer (DDS) analyzer. Because impedance is a known value, the GF can be readily obtained. And the electrical conductivity calibration equation can be shown as:

2 $${\rm{Gain}}\,\mathrm{Factor}=\,(\frac{{\rm{Admittance}}}{{\rm{Code}}})=(\frac{1/\mathrm{Impedance}}{{\rm{Magnitude}}})$$

The developed portable sensing system detects the electrical conductivity parameters of a liquid by discrete Fourier transform (DFT) to determine the real- and imaginary-number parts of the signal. The obtained admittance signal can be calculated by the MCU. When the designed electrodes perform a test, a temperature sensor is necessary for the developed system to convert and normalize the measured reading to the readings taken at 25 °C. Therefore, the design of the temperature sensor employs a resistor for humidity measurement and negative temperature coefficient (NTC) for temperature measurement. The temperature sensor has an 8-bit MCU, which transmits the measured temperature and humidity readings as digital signals. The temperature calibration equation is shown as follows:

3 $$K=\frac{{{\rm{k}}}_{{\rm{m}}}}{{\rm{1}}+{\rm{0.0191}}({\rm{t}}-{\rm{25}})},$$

where K (Ω/cm) represents the converted electrical conductivity (at 25 °C) and k_m_ represents the electrical conductivity measured at t°C. In this study, impedance was obtained by applying Eq. (2), after which Eq. (3) was applied with the temperature value to determine the electrical conductivity. Using the device, the data showed that the correlation coefficient was up to 0.99 between the electric signal and 0–20 mM NaCl solution. The purchased standard NaCl conductivity solution (442–1000; Myrol L company, USA) exhibited a conductivity of 1417 μS at 25 °C and was used to establish a conductivity conversion to develop the portable sensing system. And the standard conductivity solution was diluted with DI water and prepared at concentrations of 283.4, 354.3, 472.3, 708.5, and 1417 μS/cm.

### Electrodes and sensor performance in the protein factor interference test

Bovine serum albumin BSA (Sigma-Aldrich, USA) was used as an interfering protein factor. BSA protein was spiked at concentrations of 3.5, 7.0, 10.0, 15.0, and 20.0 mg/mL in fixed 5 mM NaCl electrolyte solution. Samples were measured using commercial probes and co-planar electrodes with a gap length of 100 μm, including microfabricated and PCB electrodes as shown in Fig. 1. Commercial probes were purchased from Ruosull Technology (Catalogue type: DJS-1, China) and were vertically aligned. The sample size for measurements was 100 μL for the proposed electrode and 3 mL for the commercial probe. All test tubes were prepared in triplicate. Four concentrations of saliva from healthy adults were further measured: nondiluted and 2×, 3×, 4×, and 5× diluted with DI water. The conductance of all concentrations of saliva was detected using the LCR meter and 100 μm gap-length electrode over 15 min. The performance of the portable sensing system was assessed using standard NaCl conductivity solutions, which were diluted with DI water to concentrations of 0, 283.4, 354.3, 472.3, 708.5, and 1417 μS/cm at 25 °C. Measurements were performed using the portable sensing system with proposed co-planar electrodes with a 100-μm gap length.

### Clinical study

All saliva samples used in this study were from a trial entitled “Correlation between saliva conductivity and osmolality, BUN/creatinine in blood”. This trial was approved by Chang Gung Medical Foundation Institutional Review Board (approval number: 201801708B0), and all participants provided written informed consent. The study was conducted in accordance with Helsinki Declaration. Clinical blood and saliva specimens of healthy adults and farmers were collected at least 1.5 h after participants had eaten a meal, and specimens from patients with CKD were collected before their hemodialysis process. The farmers were invited to participate after finishing work. Venous blood and saliva samples were acquired from healthy office workers (N = 10), healthy farmers (N = 10), and patients with CKD (N = 10). All tested subjects sat quietly in the hospital examination room in a constant-temperature condition. The venous blood specimens were collected directly into 3-mL Vacutainer PST tubes containing spray-coated lithium heparin and gel for serum separation (BD, USA). Blood specimens were analyzed for serum osmolality using a freezing point depression osmometer (Advanced Model 3250 Osmometer, USA) and BUN and creatinine were analyzed using a chemistry analyzer (Beckman DXC880i, USA). All saliva specimens were collected before each subject swallowed and emptied their mouth. Each subject was asked to place a sterile 6-inch cotton swab (diameter = 0.9 cm, length = 15.24 cm) under his or her tongue for 5 min. The collected swab was placed in a 15 mL centrifuge tube immediately without contacting the tube opening before being stored at 4°C. The 15 mL centrifuge tube containing the cotton swab was subjected to centrifugation at 3500 rpm for 5 min, and then the saliva at the bottom was collected. And 50 μL of the collected saliva samples was diluted five times with DI water and then analyzed for saliva conductivity using the developed portable sensing system and PCB electrode.

## References

[1] Levin A (2017). Global kidney health 2017 and beyond: a roadmap for closing gaps in care, research, and policy. The Lancet.

[2] Nanayakkara, S. et al. The presence of dehydration in paddy farmers in an area with chronic kidney disease of unknown aetiology (CKDu). *Nephrology*, 10.1111/nep.13605 (2019). 10.1111/nep.13605 31099943

[3] Wegman DH (2018). Intervention to diminish dehydration and kidney damage among sugarcane workers. Scandinavian Journal of Work, Environment & Health.

[4] Nerbass FB (2017). Occupational heat stress and kidney health: from farms to factories. Kidney International Reports.

[5] Clark WF (2016). Hydration and chronic kidney disease progression: a critical review of the evidence. American Journal of Nephrology.

[6] Cheuvront SN (2010). Biological variation and diagnostic accuracy of dehydration assessment markers. The American Journal of Clinical Nutrition.

[7] Campuzano S (2019). Antifouling (Bio) materials for Electrochemical (Bio) sensing. International Journal of Molecular Sciences.

[8] Muñoz C (2013). Assessment of hydration biomarkers including salivary osmolality during passive and active dehydration. European Journal of Clinical Nutrition.

[9] Yoshizawa JM (2013). Salivary biomarkers: toward future clinical and diagnostic utilities. Clinical Microbiology Reviews.

[10] Jasim H (2018). Saliva as a medium to detect and measure biomarkers related to pain. Scientific Report.

[11] Farah R (2018). Salivary biomarkers for the diagnosis and monitoring of neurological diseases. Biomedical Journal.

[12] Wang J (2015). Salivary biomarkers of oxidative stress: a critical review. Free Radical Biology and Medicine.

[13] Anuradha BR (2015). Oral and salivary changes in patients with chronic kidney disease: A clinical and biochemical study. Journal of Indian Society of Periodontology.

[14] Bagalad BS (2017). Diagnostic accuracy of salivary creatinine, urea, and potassium levels to assess dialysis need in renal failure patients. Dental Research Journal.

[15] Singh M (2015). Effect of long-term smoking on salivary flow rate and salivary pH. Journal of Indian Association of Public Health Dentistry.

[16] de Almeida PDV (2008). Saliva composition and functions: a comprehensive review. The Journal of Contemporary Dental Practice.

[17] Fortes MB (2015). Is this elderly patient dehydrated? Diagnostic accuracy of hydration assessment using physical signs, urine, and saliva markers. Journal of the American Medical Directors Association.

[18] Dimeski G, Barnett RJ (2005). Effects of total plasma protein concentration on plasma sodium, potassium and chloride measurements by an indirect ion selective electrode measuring system. Critical Care and Resuscitation.

[19] Dimeski G (2010). Ion selective electrodes (ISEs) and interferences – a review. Chinica Chimica Acta.

[20] Gupta BL (1978). Electron microprobe and ion-selective microelectrode studies of fluid secretion in the salivary glands of calliphora. The Journal of Experimental Biology.

[21] Dawes C (1972). Circadian rhythms in human salivary flow rate and composition. The Journal of Physiology.

[22] Glaser J (2016). Climate change and the emergent epidemic of CKD from heat stress in rural communities: the case for heat stress nephropathy. Clinical Journal of American Society of Nephrology.

[23] Correa-Rotter R (2014). CKD of unknown origin in central America: the case for a mesoanerican nephropathy. American Journal of Kidney Diseases.

[24] Hu X, Yang W (2010). Planar capacitive sensors–designs and applications. Sensor Review.

[25] Joucla S, Yvert B (2009). Improved focalization of electrical microstimulation using microelectrode arrays: a modeling study. PloS one.

[26] Gall I (2013). The effect of electric fields on bacterial attachment to conductive surfaces. Soft Matter.

[27] Kuo Y-C (2018). Improving sensitivity of a miniaturized label-free electrochemical biosensor using zigzag electrodes. Biosensors Bioelectronics.

[28] Ye Y (2016). A novel method for proximity detection of moving targets using a large-scale planar capacitive sensor system. Sensors.

[29] Walsh NP (2004). Saliva parameters as potential indices of hydration status during acute dehydration. Medicine & Science in Sports & Exercise.

[30] Shirreffs SM, Maughan RJ (1998). Urine osmolality and conductivity as indices of hydration status in athletes in the heat. Medicine and Science in Sports and Exercise.

[31] Lamb EJ (2009). How should proteinuria be detected and measured?. Annals of Clinical Biochemistry.

[32] Lin L, Chang C (1989). Determination of protein concentration in human saliva. The Kaohsiung Journal of Medical Sciences.

[33] Vibhakar PA (2013). Salivary total protein levels and their correlation to dental caries. International Journal of Oral and Maxillofacial Pathology.

